# Hearing From Mums-to-Be: A Qualitative Study Looking at the Experience of Mothers on the PRAM Project

**DOI:** 10.1192/bjo.2025.10532

**Published:** 2025-06-20

**Authors:** Theint Sandi Win Naung, Min Sian Chua, Flora Yong, Sharon Sung, Helen Chen

**Affiliations:** 1 Duke-NUS Medical School, Singapore, Singapore.; 2 KK Women’s & Children’s Hospital, Singapore, Singapore

## Abstract

**Aims:** Antenatal depression significantly impacts maternal and foetal health outcomes, yet it remains underdiagnosed and undertreated. The Psychological Resilience in Antenatal Management (PRAM) programme at KK Women’s and Children’s Hospital in Singapore was established in December 2022 as a strategy to identify antenatal depression early among pregnant patients. Under the PRAM programme, universal antenatal depression screening is integrated into the routine care programme for pregnant patients, using a modified version of the Edinburgh Postnatal Depression Scale (EPDS) questionnaire during their routine obstetric check-up in the second trimester, for early intervention by the perinatal mental health team.

This qualitative study explores the lived experiences of pregnant women who have undergone screening and intervention under the PRAM programme. It seeks to understand their perceptions of the screening and intervention process, identify barriers and facilitators to help-seeking, and examine effective components of the therapeutic process.

**Methods:** Using an Interpretative Phenomenological Analysis (IPA) approach, semi-structured interviews were conducted with 10 women who have participated in the PRAM programme between November 2023 to January 2025. Interviews were completed either virtually over Zoom (N=8) or in person (N=2). The interviews explored participants’ experiences with antenatal depression screening, subsequent interventions, and their overall pregnancy journey while managing mental health concerns.

**Results:** Preliminary analysis reveals several key themes in participants’ experiences. For half of the participants (N=5), the screening process served as an opportunity for self-evaluation and mental health awareness. Obstetricians have also been identified to be crucial facilitators, serving as the initial point of psychiatric referral and influencing women’s decisions to seek support. A significant barrier identified by four participants was the stigma associated with psychiatric diagnoses and receiving psychiatric help. Additionally, participants emphasised the importance of spousal involvement in the therapeutic process, with several women expressing a desire for greater partner participation in their mental health journey.

**Conclusion:** Understanding women’s experiences with the PRAM programme contributes to improving screening protocols and developing more effective, patient-centred approaches to managing antenatal depression. The findings highlight the need for integrated care pathways that address stigma, enhance partner involvement, and strengthen the role of obstetricians in perinatal mental health care. These insights can inform the development of more comprehensive and accessible mental health support services within perinatal care settings.

